# Recent advances in understanding *Streptomyces*

**DOI:** 10.12688/f1000research.9534.1

**Published:** 2016-11-30

**Authors:** Keith F. Chater

**Affiliations:** 1 Department of Molecular Microbiology, John Innes Centre, Norwich, UK

**Keywords:** streptomyces, genomics, streptomyces ecology, streptomyces evolution, pathogenic streptomyces

## Abstract

About 2,500 papers dated 2014–2016 were recovered by searching the PubMed database for *Streptomyces*, which are the richest known source of antibiotics. This review integrates around 100 of these papers in sections dealing with evolution, ecology, pathogenicity, growth and development, stress responses and secondary metabolism, gene expression, and technical advances. Genomic approaches have greatly accelerated progress. For example, it has been definitively shown that interspecies recombination of conserved genes has occurred during evolution, in addition to exchanges of some of the tens of thousands of non-conserved accessory genes. The closeness of the association of *Streptomyces* with plants, fungi, and insects has become clear and is reflected in the importance of regulators of cellulose and chitin utilisation in overall *Streptomyces* biology. Interestingly, endogenous cellulose-like glycans are also proving important in hyphal growth and in the clumping that affects industrial fermentations. Nucleotide secondary messengers, including cyclic di-GMP, have been shown to provide key input into developmental processes such as germination and reproductive growth, while late morphological changes during sporulation involve control by phosphorylation. The discovery that nitric oxide is produced endogenously puts a new face on speculative models in which regulatory Wbl proteins (peculiar to actinobacteria) respond to nitric oxide produced in stressful physiological transitions. Some dramatic insights have come from a new model system for *Streptomyces *developmental biology, *Streptomyces venezuelae*, including molecular evidence of very close interplay in each of two pairs of regulatory proteins. An extra dimension has been added to the many complexities of the regulation of secondary metabolism by findings of regulatory crosstalk within and between pathways, and even between species, mediated by end products. Among many outcomes from the application of chromosome immunoprecipitation sequencing (ChIP-seq) analysis and other methods based on “next-generation sequencing” has been the finding that 21% of *Streptomyces *mRNA species lack leader sequences and conventional ribosome binding sites. Further technical advances now emerging should lead to continued acceleration of knowledge, and more effective exploitation, of these astonishing and critically important organisms.

## Introduction

The majority of antibiotics used in medicine, veterinary practice, and agriculture originate from *Streptomyces* bacteria. Genomic analysis has shown that any one strain has the potential to make tens of such secondary metabolites, and metagenomic analysis has revealed vast numbers of relevant biosynthetic gene sets ^1, 2^. These organisms are therefore being studied ever more intensively in the expectation that they will contribute significantly to the provision of new therapeutic agents to combat the global emergence of antibiotic resistance among pathogenic bacteria, as well as providing other bioactive agents with medical applications. However, this is only one aspect of the interest and importance of streptomycetes. Ecologically, streptomycetes have key roles in the natural recycling of the globally abundant cell walls of fungi and plants. Some of them have evolved intimate partnerships with insects or plants, and a few have acquired pathogenic attributes. The interplay of these associations with *Streptomyces* molecular physiology is a strong theme in this review and has been underpinned by continuing in-depth analysis of the model organism *Streptomyces coelicolor* A3(2), which has been studied genetically for about 60 years ^3^.

**Figure 1.**
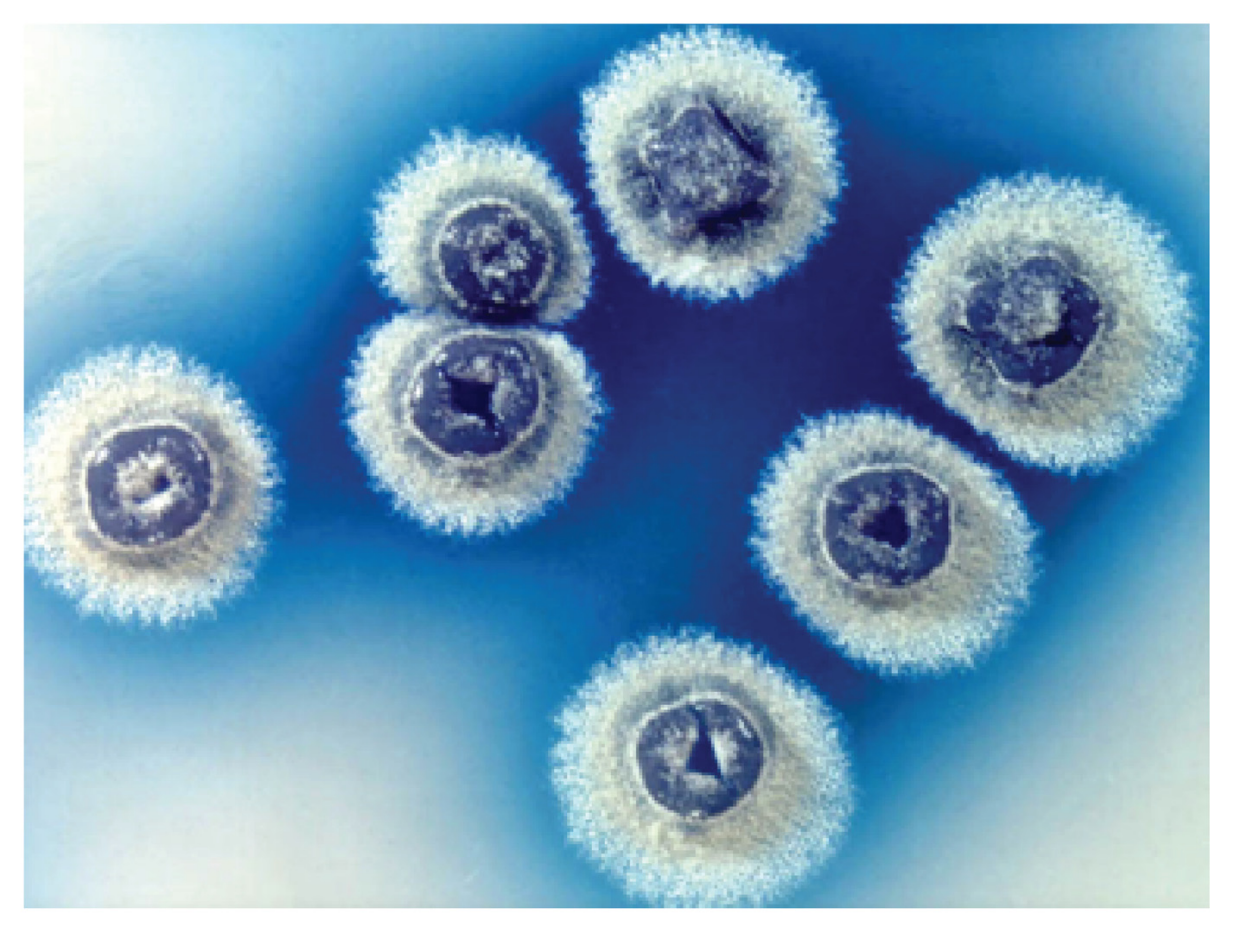
Colonies of *Streptomyces coelicolor* A3(2). The fuzzy surface of these mould-like colonies is made up of aerial hyphae carrying chains of spores (photo, K.F. Chater).

Unusually for bacteria, streptomycetes grow as branching hyphal filaments to form a mat of fungus-like mycelium, from which emerge aerial branches that bear chains of spores ( Figure 1). Very significant progress has been made recently in the analysis of this complex growth and development in both *S. coelicolor* and the emerging model species *Streptomyces venezuelae,* which sporulates very readily, even in liquid culture.

These two model organisms are providing new insights into how antibiotic production is integrated into overall physiology, taking advantage of the fact that, among the more than 20 secondary metabolites of *S. coelicolor*, two are pigmented antibiotics (the polyketide actinorhodin [ACT] and the red tripyrrole prodiginines [RED]), while *S. venezuelae* makes chloramphenicol and the polyketide jadomycin. These studies have pointed to an unexpected role for antibiotics themselves as regulatory ligands mediating autoregulation, cross-regulation, and interspecies interactions.

This wide-ranging topical snapshot of the genus has been compiled from a selection of the approximately 2,500 papers about *Streptomyces* published in 2014–2016. Where possible, I have cited review articles that provide context to the recent advances. In concentrating on whole organism physiology and evolution, I have excluded certain major *Streptomyces* research areas, notably taxonomy, systematics, and natural product chemistry.

## Ecology and evolution

### More than just soil organisms…

Streptomycetes, which are abundant in soil, are believed to have originated around 400 million years ago, when the land was being colonised by green plants. Their major role in the solubilisation of cell wall or surface components of plants, fungi, and insects ^4^ suggests that they played a part in the composting of early land plants and hence in the formation of primeval soil. This conserved ecology over evolutionary time is reflected at the genome level: for example, *Streptomyces reticuli*, which is particularly active in cellulose degradation, has an estimated 456 genes for proteins involved in the binding, degradation, and utilisation of cellulose and other complex and simple carbohydrates ^5^. *S. coelicolor* also has eight genes for cellulase-like proteins, at least one of which is a true cellulase, even though *S. coelicolor* cannot grow with cellulose as its sole carbon source ^6^. This situation is widespread among streptomycetes, prompting speculation that most isolates have evolved to live in mixed, cooperating communities, within which only a few species are competent to release amenable products from native cellulose ^7^. In addition, streptomycetes have had a long evolutionary association with fungi, with fungal cell walls being a key source of nutrition; thus, *S. coelicolor* has (at least) 13 chitinases regulated by the important global regulator DasR, which coordinates many aspects of *Streptomyces* biology in response to the chitin-derived ligands N-acetylglucosamine or N-acetylglucosamine-phosphate ^4, 8– 10^, as well as two chitosanases and a dedicated oligoglucosamine uptake system that are regulated independently of DasR ^11^.

Reinforcing their close association with plants (reviewed in 12), streptomycetes are abundantly represented in metagenomic analysis of the rhizosphere ^13^, and they are among the most frequent endophytes: a search of PubMed for “Streptomyces endophyte” yielded 74 papers published in the last 15 years, with 38 dated 2013–2016. Many studies have focused on endophytes from traditional medicinal plants, often in the hope that the endophytes may be responsible for the medicinal properties (e.g. 14– 19). Indeed, *Streptomyces* endophytes produce various antagonists of plant disease agents and pests such as bacteria, fungi, and insects ^20^, consistent with potentially ancient adaptive benefits that might be turned to agricultural and medical use in the modern world. Streptomycetes may also stimulate plant growth ^21, 22^, sometimes by producing phytohormones (e.g. 23, 24). Endophytic streptomycetes are also found in diverse non-medicinal dicot plants ^25, 26^ and in monocots including rice ^22^, suggesting (but not proving) that such interactions may date back hundreds of millions of years. Metagenomic evidence of particularly exuberant diversity of soil streptomycetes at low latitudes ^27, 28^ might reflect greater plant and insect diversity in the tropics (along with some degree of specificity in interactions between *Streptomyces* and plants or insects).

Streptomycetes are partners in other mutualistic interactions involving plants. Inoculation of the rhizosphere of rooted oak cuttings with *Streptomyces* sp. AcH 505 not only stimulated mycorrhizal development but also elicited plant defence mechanisms, leading to increased resistance to powdery mildew infection ^29^. Streptomycetes are also carried by ants that cultivate black food yeasts inside cavities (“domatia”) in host plants. In this mutualism, the plants secrete sugary exudates to the ants and yeasts, while the ants ward off predators and generally clean up their host. The streptomycetes produce inhibitors of other, unwanted, fungi that might invade the domatia ^30^. In a broad survey, the streptomycetes involved in these plant–ant interactions fell into just four distinct taxa, while many other taxa were unrepresented, implying specific adaptations ^30^. Other cases of insect-borne streptomycetes preventing unwanted fungal invasion have been extensively reported in the last 20 years, such as in the cases of leaf-cutter ants and beewolf wasps ^12^: in the latter case, some degree of co-evolution of hosts and microbes has been taking place for perhaps 68 million years ^12, 31^. Streptomycetes isolated from associations with diverse arthropods that feed on plant biomass are also highly enriched for two taxa having the ability to degrade cellulose completely, compared to random soil isolates, suggesting that this ability is valuable in the symbiosis ^7^.

The production of antibiotics by streptomycetes has long suggested that they may be formidable competitors in natural environments, but it was a surprise that, when growing on agar spread with other bacteria, the growing hyphal tips of most strains caused local lysis of what can reasonably be interpreted as their prey ^32^. Such predatory activity may add a new perspective to the benefits of streptomycetes to plants and insect mutualistic partners.

In a further illustration of the ecological diversity of streptomycetes, strains adapted to marine conditions are prominent in the microbiota of marine sponges ^33^.

### Streptomycetes as pathogens: evolution and regulation of potato scab

Not all interactions of streptomycetes with complex eukaryotes are beneficial. Scab disease of potato and other tuber/root crops is caused by several different streptomycetes and (as in many cases of bacterial pathogenesis) involves a laterally acquired pathogenicity island that presumably evolved with the emergence of scab hosts, which are all flowering plants that evolved long after simpler plants had colonised the land. In *Streptomyces turgidiscabies*, this entire DNA island can excise from the host genome and transfer into a new host, where it can again integrate via a site-specific recombinase at the palindromic sequence 5′-TTCATGAA-3′ ^34^. The presence of internal copies of this sequence can lead to modular excision of the island. Fixation of the pathogenicity genes in some other potato scab agents ( *Streptomyces scabies* and *Streptomyces acidiscabies*) is correlated with degradation of the recombinase gene and/or its target sites ^34^ or with loss of a module that encodes all the functions needed for mobilisation ^35^.

Remarkably, the expression of some of the pathogenicity genes, including those for the phytotoxin thaxtomin, is determined in part by the cellobiose sensor CebR, the universal regulator of *Streptomyces* cellulolytic genes, integrating pathogenicity-linked cellulolytic activity with more ancient overall responses to cellulose-related metabolites ^36^. The connection of pathogenicity with cellulose is further emphasised by the fact that thaxtomin is an inhibitor of plant cellulose synthase. The biosynthetic clusters for thaxtomin and a second phytotoxin, coronafacoyl phytotoxin, are under further control from pathway-specific regulatory genes that appear to respond to plant metabolites ^37^ and from global regulatory genes ( *bld* genes that regulate colony differentiation and antibiotic production, see below) ^38^. In an extension of this regulatory scenario, the induction of a large swathe of glycohydrolytic enzymes of *S. scabies* is enhanced when cellulose in the medium is supplemented with suberin, a complex lipid- and polyaromatic-containing major component of the potato tuber periderm ^39^.

Although scab research has focused on the pathogenicity islands, comparative genomics has revealed 64 other genes that are common to four distinct scab agents but absent from non-pathogens, as well as an exceptionally high proportion of enzymes such as pectinases and cutinases that would aid or take advantage of pathogenicity ^35, 40^.

### Genome and pan-genome evolution, the role of recombination, and its molecular basis

Comparative genomics has been dramatically accelerated by the advent of cheap and rapid sequencing (but note that the quality of the deposited *Streptomyces* genome sequences is variable ^41^). Early deductions about the core and accessory genes of streptomycetes have been refined ^42^: the genomes of 17 diverse strains (ranging from 6.7 to 12.3 Mb) contained from 5,382 to 10,022 CDSs, of which 2,018 were universally present (the core genome, which occupies most of the central region of *Streptomyces* linear chromosomes), with most of the remaining genes being present in only one or a few genomes. This accessory genome totalled 32,574 genes, so the *Streptomyces* pan-genome comprises at least 34,592 genes. The accessory genes are mostly clustered at the chromosome ends, which are well known to be unstable and subject to replacement by recombination with incoming linear plasmids or conjugally transferred segments of chromosomes from other streptomycetes, but recent analyses have revealed an unexpectedly high level of historical recombination within representative core genes too, resulting in phylogenetic incongruence among these genes ^40, 43, 44^. The recombination levels detected are high enough to raise the question of whether the evolutionary radiation of the genus has taken place over a significantly shorter time than has been widely accepted ^44^. During divergence from common ancestors, such genetic exchange continued at a rate that decreased as divergence increased, generating a reticulate origin of extant streptomycetes, largely defined by a relatively small number of ancestral recombination events. Recombination associated with nascent divergence continues into the near-present, since recombination events detected by genome analysis of independent isolates of the same species are about 100-fold more frequent than those between species ^43^. The fixation of particular recombination events is thought to reflect periods of rapid demographic expansion ^27, 28^.

**Figure 2.**
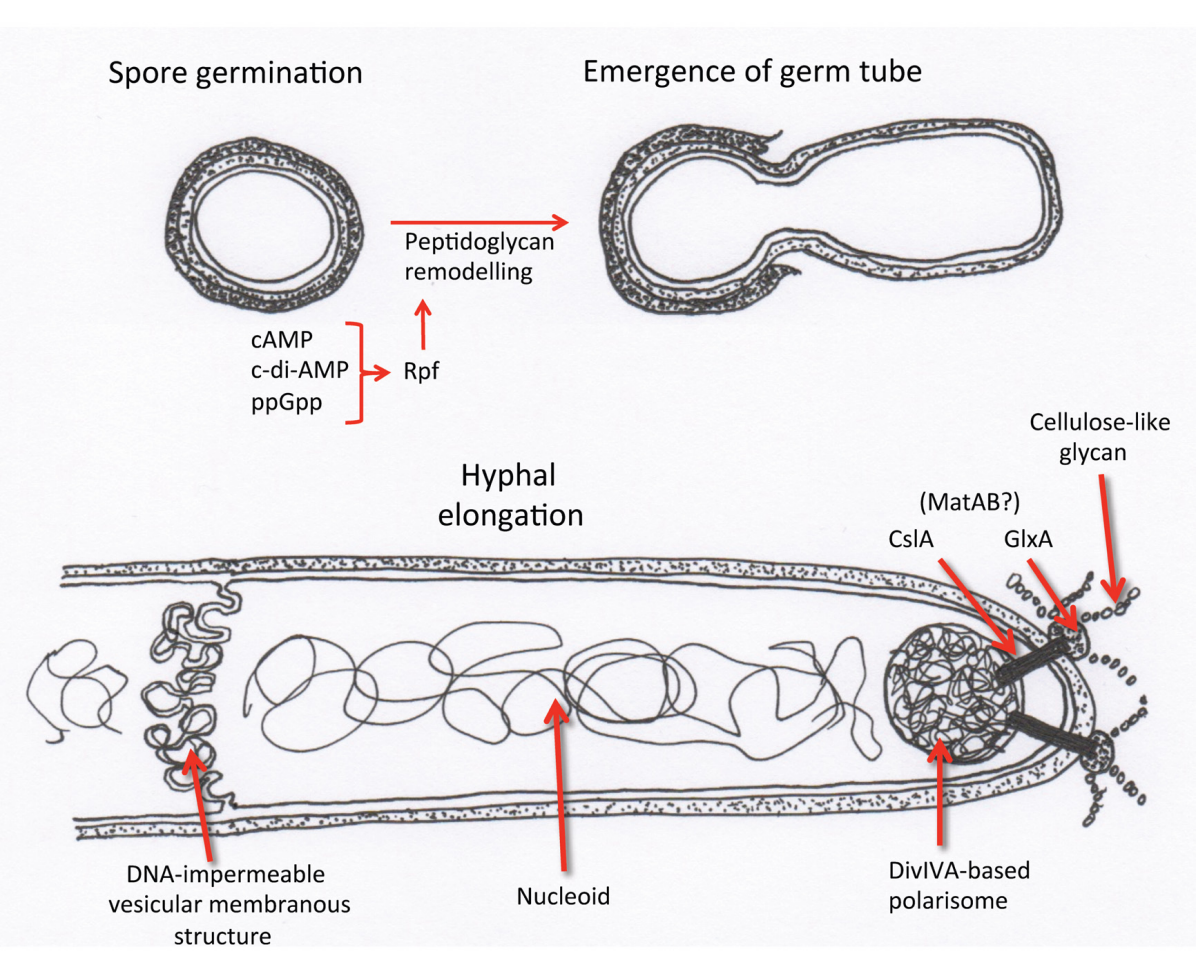
Recent discoveries about germination and hyphal growth. Three different nucleotide signalling molecules are involved in stimulating the production of peptidoglycan hydrolase(s) (Rpf, for resuscitation-promoting factors), leading to remodelling of the spore wall and the emergence of a germ tube ^49^. Continued hyphal elongation is co-ordinated by the DivIVA-based polarisome ^50^ and (in addition to cell wall growth) involves the extracellular production of cellulose-like glycan catalysed by polarisome-linked ClsA (cellulose synthase-like) associated with the copper oxidase GlxA ^52, 53^. MatAB proteins have an uncharacterised role in glycan production ^54^. Vesicular membrane structures form in apparently irregular locations within hyphae, and some of them extend across the hyphal compartment, separating nucleoids ^57, 58^.

The high frequency of genetic exchange implied by these studies may be because conjugal DNA transfer in streptomycetes is much more efficient than it is in most other bacteria, at least in the laboratory: instead of a complex type IV secretion apparatus transferring single-stranded DNA, streptomycetes employ the products of *traB* genes encoded by autonomous or chromosomally integrated plasmids to transfer double-stranded DNA ^45^. TraB proteins belong to the FtsK/SpoIIIE family needed in many bacteria to complete the segregation of chromosomes into newly formed daughter cells, but, while FtsK/SpoIIIE proteins assemble in the nascent septum, TraB proteins assemble at growing hyphal tips. Transfer, which is ATP dependent, involves the interaction of TraB with short recognition sequences (consensus 5′-GACCCGGA-3′ in plasmid pSVH1): 25 clusters of four such sequences are also spaced along the chromosome of *S. coelicolor*, accounting for chromosome mobilisation ^45^. Initial plasmid transfer is followed by the remarkable spread of the plasmid, sometimes throughout most of the recipient mycelium, in a process in which specialised Spd proteins assemble with TraB in the vegetative septa that occur roughly once every 10–20 μm ^45, 46^ ( Figure 2). The importance of TraB for plasmid spread (presumably providing the ATPase-driven motor function lacking from Spd proteins) was nicely demonstrated by showing that the presence of TraB in a recipient permitted efficient spreading following the occasional transfer of a *traB*-deleted plasmid ^46^.

It was a surprise when a recent cytological study showed that plasmid invasion events were associated with contact between the lateral walls of donor and recipient hyphae, and events involving donor tips were not seen ^46^. This unexpected result may still be compatible with tip involvement: TraB may also assemble at nascent branch tips, some distance away from leading tips. In any case, the cytological analysis could presumably detect only transfer events that had occurred a significant time earlier, after subsequent plasmid multiplication and spread had already occurred, so the state of hyphae at the time of primary transfer could not be assessed.

### Within-species comparative genomics

Generally, comparative genomics of closely related streptomycetes has had little attention, though marine isolates from two distinct sponges were interestingly closely related to the terrestrial *Streptomyces albus* G derivative J1074 (which is often used for heterologous expression work) in a study identifying candidate marine-adaptation genes present only in the two marine isolates and in some other marine actinomycetes ^33, 47^. Four further J1074-related organisms were subsequently found in 140 *Streptomyces* genomes available at the time, making this species group the most readily isolated to date ^33, 47^. This provided an opportunity to examine the dynamics of acquisition and loss of secondary metabolite biosynthetic gene sets in relatively recent evolution (i.e. within a species group). Ten biosynthetic gene clusters were specific to, and conserved among, the J1074 group, while a roughly equal number showed some degree of isolate specificity ^33, 47^.

## New perspectives in growth and development of *Streptomyces*

### Germination is controlled by several second messengers

Spore germination involves the action of “resuscitation-promoting factors” (Rpfs, specialised peptidoglycan hydrolases), probably as part of a peptidoglycan remodelling system ^48^. At least one of five partially functionally redundant Rpfs of *S. coelicolor* is subject to multilevel regulation by different nucleotide second messengers: transcription initiation, controlled by the cAMP-binding protein previously associated with germination; transcriptional attenuation via a riboswitch responsive to cyclic di-AMP; and changing rates of proteolysis in response to levels of ppGpp ^49^ ( Figure 2).

### Importance of cellulose-like glycan synthesis for hyphal growth

*Streptomyces* growth is unusual, but not unique, among bacteria in taking place at tips through the activities of a protein complex (“polarisome”). The key polarisome component, DivIVA, coordinates aspects of intracellular and cell surface growth in ways that may differ during vegetative and reproductive growth (for reviews, see 50, 51). It is emerging that normal growth and development require deposition at hyphal tips of an uncharacterised glycan through the combined action of a cellulose synthase-like protein (ClsA) and GlxA, the product of the gene downstream of *clsA*, which is a copper oxidase that may oxidise the glycan as it is secreted ^52^ ( Figure 2). Like ClsA, GlxA is tip-located at least at some growth stages ^53^, though the two proteins have not been analysed together. The synthesis of extracellular polysaccharides soon after germination, under the control of *clsA*, *glxA*, and the newly identified locus *matAB*, is responsible for the aggregation of germlings, leading to the formation of mycelial clumps in submerged culture, a well-known problem in industrial fermentations ^54, 55^.

### Membranous structures appear to subdivide the cytoplasm of vegetative hyphae into compartments

The recent application of advanced microscopic methods has suggested that vesicular, DNA-impermeable membranous structures may delimit compartments in vegetative hyphae in the absence of conventional septa, may account for the viability of some hyphal fragments of *ftsZ* mutants, and may be implicated in the heterogeneous staining of vegetative hyphae with vital stains (interpreted as programmed death ^56– 58^) ( Figure 2). These intriguing observations raise questions of how the structures are placed in time and space, how they affect the spread of plasmids through the mycelium (see above), and whether they might limit the spread of initially tip-localised phage infections. Addressing such questions may require inventive genetic approaches.

### Complex and subtle influences on the initiation of aerial development, involving transcription, proteolysis, and hormone-like molecules

The onset of reproductive aerial growth is sensitive to diverse stresses and signals. In *S. coelicolor*, most of the nine σ ^B^-like sigma factors (compared to four in *Bacillus subtilis*) are involved in stress responses and/or differentiation. There is growing evidence of complex crosstalk interactions among the even larger number of anti-sigma and anti-anti-sigma factors for this class of σ factor, and it has been suggested that the signal input to regulate these interactions may involve some of the variable, but always multiple, collection of *whiJ*-like gene sets present in *Streptomyces* genomes: the clusters include anti-sigma genes of this type and have been implicated in development and antibiotic production ^51, 59^. In one such cluster, which is known to influence antibiotic production and differentiation, a small RNA antisense to the Sco4676–4677 intergenic region has been found to have complicated regulatory effects on Sco4676–4677 expression ^60^.

Furthermore, particularly since the discovery that actinomycetes have a eukaryotic-like proteasome system, with ubiquitinylation of targets being replaced by pupylation ^61^, regulation at the level of protein degradation is becoming increasingly apparent. This has been reinforced by the cataloguing of pupylation/proteasome targets and the phenotypes of mutants in the system, which show changes in differentiation and antibiotic production levels ^62– 64^. Presumably, controlled proteolysis is involved in the death of some hyphal compartments that accompanies stages of growth and development ^56^.

It has been known for decades that in *Streptomyces griseus* the repression of a central developmental regulatory gene, *adpA*, by the TetR-like protein ArpA is relieved by the accumulation of A-factor, an extracellular hormone-like gamma-butyrolactone (GBL). It now seems that *adpA* in most, perhaps all, other streptomycetes may be subject to regulation by similar ArpA-like repressors, but with species-specific GBL ligands, permitting coordinated sporulation of one species without triggering sporulation of nearby different species ^65^. In an opposite scenario, *S. coelicolor* and *S. venezuelae* produce an identical GBL called SCB1 and SVB1, respectively, making it possible for them to respond to each other ^66, 67^.

### Spectacular insights using a new model organism, *Streptomyces venezuelae*: time-lapse cell biology, heterodimeric regulatory proteins, global regulation by a cyclic di-GMP receptor, and developmental differences between species

*Streptomyces* colony development has usually been studied on solid agar medium, since *S. coelicolor* (like many other streptomycetes) does not undergo a full developmental cycle in liquid culture. Studies of surface-grown cultures are bedevilled by developmental asynchrony and heterogeneity, which are particularly problematic for high-resolution biochemical analysis, including “omic” approaches to the definition of the regulons controlled by developmental regulatory genes. These problems have been circumvented by the adoption of *S. venezuelae*, which grows very rapidly and undergoes comprehensive and near-synchronous differentiation in liquid culture, including under coverslips ^68^. In a first illustration of the value of this system, a detailed time-lapse study has shown that the ParA and ParB chromosome segregation proteins play an important coordinating role in the transition from ongoing aerial growth to growth cessation and the onset of sporulation septation, and even have opposing influences on the rate of tip extension and eventual length of pre-sporulation hyphae ^69^. Further advances are promised by the development of a microfluidics system that allows continuous direct observation of fluorescently marked key proteins through the entire developmental cycle ^68^.

Submerged sporulation has allowed the highly successful combined application of microarray-based transcriptomics and chromosome immunoprecipitation sequencing (ChIP-seq) analysis to reveal two exciting and novel cases of very close interactions between different developmental regulators. In one case ^70^, an atypical response regulator, BldM, has two rounds of activity: first, it forms a homodimer to activate other developmental regulatory genes and morphogenetic functions needed for aerial hyphae to grow and, second, it forms a heterodimer with another atypical response regulator, WhiI, to activate genes needed for the aerial hyphae to turn into spore chains. In another case ^71^, with wide implications for all actinomycetes including pathogenic mycobacteria, two enigmatic proteins conserved across all actinomycetes have been found to bind to the same target promoters, activating some and repressing others. These proteins are WhiA, which is also present in Gram-positive Firmicutes and has a structure closely similar to that of eukaryotic homing endonucleases (but lacking key catalytic residues), and WhiB, the archetype of the large “Wbl” family of iron-sulphur proteins represented throughout actinomycetes but found nowhere else (other Wbl proteins conserved among streptomycetes include two other developmental regulators, WblA and WhiD) ^51^. The ChIP-seq/transcriptome profiles of targets of Bld and Whi regulators in *S. venezuelae* have provided many clues about the nature and coordination of the components contributing actively to development ^70– 72^.

It is likely that BldM, WhiI, WhiA, and WhiB respond to different signal inputs, and the elucidation of these signals is a major challenge (but see the next section). These four developmental activators, and most of the well-known developmental regulatory *bld* and *whi* genes and many of their targets, are among the large number of genes directly controlled by BldD ^72^, a master repressor of development whose regulatory ligand has been revealed as cyclic di-GMP ^73^. This important discovery begins to provide an explanation for the presence of eight actual or predicted diguanylate cyclase genes in *S. coelicolor,* some with associated cyclic di-GMP phosphodiesterase domains ^74^. Notably, the [BldD] _2_:[cyclic di-GMP] _4_ complex is structurally new to science ^73, 75^.

Although it is highly probable that the developmental programmes of all streptomycetes are broadly the same, some of the details may differ between species. This seems to be the case with *whiG*, which encodes a sigma factor important in initiating sporulation septation of aerial hyphae. Although *whiG* transcription depends on WhiA and WhiB in *S. venezuelae* ^71^, in *S. coelicolor* its transcription was nearly the same in *whiA* or *whiB* mutants as in the wild-type ^76^. In another example, the promoter of the WhiI-dependent *inoA* gene of *S. coelicolor* (for inositol phosphate synthetase) could bind truncated WhiI *in vitro* ^77^, indicating that WhiI could bind DNA in the absence of BldM; yet in ChIP-seq analysis of an *S. venezuelae bldM* mutant, WhiI did not bind to any targets ^70^. Other inter-species differences were previously noted between *S. coelicolor* and *S. griseus* ^78^. A different kind of surprise concerning the WhiG sigma was the discovery in *Streptomyces chattanoogensis* that it can bind to target promoters in the absence of RNA polymerase core enzyme, including some in the gene cluster for natamycin production: binding was either to typical WhiG-binding sites or to CGTCA repeat elements, and was needed for gene activation ^79^.

### Could endogenous nitric oxide have an important developmental regulatory role?

Speculation about a possible role of nitric oxide as a regulatory ligand for WhiB and other Wbl proteins was stimulated by the extremely high affinity for nitric oxide of some Wbl proteins of streptomycetes and mycobacteria, which has been studied in detail at the molecular level ^80– 83^. It has generally been implied that the source of nitric oxide would be exogenous (e.g. produced by eukaryotic hosts as a defence mechanism). However, comparative genomics revealed the general presence in Wbl-containing actinomycetes of other nitric oxide-interacting proteins (or biochemically unstudied homologues) that might be involved in cycling nitric oxide, raising the possibility that *endogenous* nitric oxide might regulate development by interacting with Wbl proteins ^51^.

**Figure 3.**
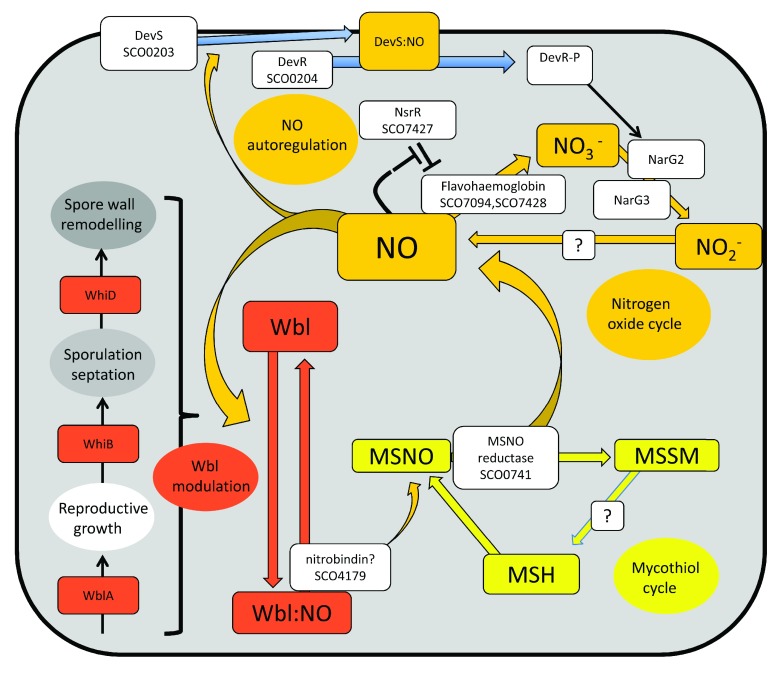
Model integrating the nitrogen oxide cycle (ochre) with mycothiol cycling (yellow) and with morphological development mediated by Wbl proteins (red) in *Streptomyces coelicolor*. In the nitrogen oxide cycle ^84^, nitrate generated intracellularly by an unknown pathway is reduced to nitrite mainly by the NarG2 enzyme ^87^. Nitrite is the source of nitric oxide (NO) (mechanism unknown) ^84^. Nitric oxide is oxidised to nitrate by flavohaemoglobins specified by SCO7428 (incorrectly given as SCO7472 in 84) and SCO7094, which are induced when nitric oxide binds to NsrR, the repressor of both genes ^84, 90^. Internal nitric oxide also binds the haem-containing sensor kinase DevS, which then phosphorylates the cognate response regulator DevR. DevR-P induces Nar2 in an autoinducing feedforward loop ^84^. Nitric oxide binds strongly to Wbl proteins such as WblA, WhiB, and WhiD, modulating their regulatory activity ^81^ and hence coordinating differentiation. Other regulatory components may take part in the action of Wbl proteins: for example, WhiB target genes are all also dependent on WhiA protein ^71^. It is speculated that the Wbl proteins are denitrosylated by the SCO4179 (nitrobindin?)-mediated transfer of nitric oxide to mycothiol (MSH) to generate MSNO ^51^. MSNO is denitrosylated by MSNO reductase, with the resulting oxidised mycothiol then being reduced to MSH by an undetermined mechanism.

This model was compromised by the absence of evidence for the conserved endogenous production of nitric oxide across actinomycetes, most of which do not have a conventional nitric oxide synthase. However, endogenous nitric oxide production involving conserved actinobacterial genes has now been convincingly demonstrated in *S. coelicolor* ^84^ and mycobacteria ^85, 86^ ( Figure 3). Thus, in *S. coelicolor*, organic nitrogen is converted by an unknown route to nitrate, which is reduced by nitrate reductase to nitrite ^84^. This mainly involves NarG2, one of three nitrate reductases in *S. coelicolor*: NarG2 is predominant in growing hyphae and is induced by moderate hypoxic downshift, while NarG3 has a subordinate role and NarG1 is mainly active during germination ^87^. Nitrite was shown in turn to be the source of nitric oxide (albeit again by an unknown route) ^84^. This source of nitric oxide mirrors a pathway important in eukaryotes ^88^, where endogenous nitric oxide is a major physiological signalling molecule, even influencing epigenetic changes ^89^.

The recycling of endogenous nitric oxide was also studied by 84 and is illustrated in Figure 3, which also shows how recycling might be connected to the regulatory functions of Wbl proteins. Nitric oxide re-enters the nitrogen oxide cycle via flavohaemoglobin, which oxidises nitric oxide to nitrate. In a feedforward mechanism, nitric oxide is the ligand of NsrR, which represses the *hmpA1* and *hmpA2* genes for flavohaemoglobin and its own promoter ^84, 90^. Remarkably, the affinity of NsrR for the *hmpA* promoters is more sensitive to nitric oxide than is its affinity for the *nsrR* promoter ^91^, permitting different strengths of induction of flavohaemoglobin as NO concentration increases. Another dimension of the cycle is provided by the interaction of endogenous nitric oxide with DevS, a protein kinase that activates the cognate response regulator DevR, which in turn activates the *narG2* gene for the major nitrate reductase needed for nitric oxide production ^84^. Interestingly, an earlier report ^92^ found that DevS could also phosphorylate the SCO3818 response regulator, which is very similar to DevR, and might therefore also be involved in regulating the nitrogen oxide cycle: orthologues of SCO3818 are very widespread across actinobacteria, whereas DevS orthologues are more sporadic across the phylum ( http://streptomyces.org.uk/actinoblast/).

Artificially engineered production of endogenous nitric oxide triggers the onset of production of at least one secondary metabolite (undecylprodiginines [RED]) by increasing the expression of the major RED pathway activator gene *redD*, but it inhibits the onset of aerial growth ^84^. It remains to be determined whether this is a straightforward reflection of the normal signalling activity of nitric oxide or whether the experimental procedures disrupt the normal balance of the nitrogen oxide cycle. In a further twist, it appears that endogenous nitrite can also serve as an extracellular signal to nearby mycelium ^84^.

## Regulation of septum positioning and cell wall changes during the formation of spores

**Figure 4.**
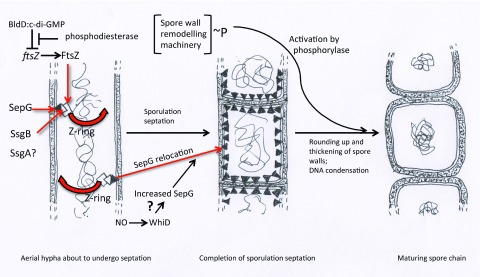
Schematic diagram of recent discoveries about late stages in sporulation. As aerial hyphae approach sporulation, c-di-GMP phosphodiesterase releases *ftsZ* from repression by BldD:c-di-GMP ^72, 74, 75^. FtsZ attaches to SsgB, held at regularly spaced positions by membrane-bound SepG ^93^. Another SsgB-like protein, SsgA, also plays a role in SsgB location, which is less well defined ^139^. FtsZ then condenses into Z-rings, which guide septation, and SepG relocates to the periphery of the prespore compartments ^93^. The spore wall remodelling complex, held in an inactive phosphorylated state, is activated by one or more of many phosphorylases ^94^ and, apparently with the involvement of SepG, causes prespores to become rounder and thicker walled. I tentatively suggest that the increased amounts of SepG at this stage may depend on the Wbl-type regulator WhiD (possibly influenced by nitric oxide [NO] – see Figure 3), since *sepG* and *whiD* null mutants have very similar phenotypes ^93, 95^.

Many of the processes involved in sporulation septation and spore maturation require high levels of proteins that might interfere with growth if expressed inappropriately, so mechanisms to prevent untimely expression can be expected. Thus, production of some of these proteins, such as FtsZ, is repressed by the BldD:cyclic di-GMP complex ^72^. Timely increased expression of such division genes, presumably triggered by cyclic di-GMP hydrolysis, must also be accompanied by a mechanism for the regular positioning of sporulation septa. New evidence suggests that SepG, a small membrane protein encoded by a gene next to *divIVA* in many Gram-positive bacteria, provides the most basic anchor yet described for the building of sporulation septa, recruiting protein SsgB, which in turn recruits FtsZ ^93^ ( Figure 4). After septation, SepG relocalises to the prespore periphery, where it may interact with the cell wall synthesising complex during spore wall remodelling. Five protein kinases, encoded by a cluster of adjacent genes, phosphorylate key proteins of this complex, keeping them inactive until sporulation septation begins: it is assumed that activation involves one or more of the more than 50 phosphatases encoded by the *S. coelicolor* genome ^94^.

The variable size, thinner walls, diffuse nucleoids, and heat sensitivity of *sepG* mutant spores are intriguingly reminiscent of the phenotype of mutants in the conserved *whiD* gene ^95^, which encodes a Wbl protein whose nitric oxide interactions have been particularly closely studied (e.g. 83 [see above]). Conceivably, *sepF* could be an important WhiD target ( Figure 4).

### Other aspects of cellular biochemistry that change during growth and development: lipids and glycogen

In a new perspective on development, the lipid composition of *S. coelicolor* differed strikingly when comparing cultures grown on solid or in liquid media, and mutants defective in ornithine lipid biosynthesis showed precocious development and antibiotic production ^96^. It would be of interest to apply such an analysis to *S. venezuelae*.

Studies of carbon storage metabolism in *Streptomyces* and mycobacteria had revealed a new and widespread pathway for glycogen biosynthesis ^97, 98^. This GlgE pathway is duplicated in most streptomycetes, with the two sets being expressed differently during development, and the pathway generally exists alongside the classical GlgAC pathway. In an exception to this generalisation, *S. venezuelae* has only one *glgE* gene set and lacks *glgAC*. This made it possible to construct glycogen-free mutants, leading to the finding that spores could be generated without the developmental accumulation of glycogen. Sporulation appeared to be normal, unless the strain/medium combination resulted in the accumulation of the toxic GlgE substrate maltose-1-phosphate ^98^.

## Stress responses and secondary metabolism

### Connecting redox homeostasis to primary metabolism

Global stress responses often involve the action of alternative sigma factors, whose target regulons may be informative in understanding physiological and cell biological adjustments that have evolved to accommodate stresses. Thus, it was recently found that one SigR target, the *ndgR* gene, encodes a regulator of sulphur assimilation and branched-chain amino acid synthesis. NdgR is presumed to affect redox homeostasis at the levels of precursor and cofactor supply to redox buffering agents such as mycothiol and supply of CoA derivatives of branched-chain fatty acids for membrane biosynthesis ^99^.

### From global physiology to the expression of biosynthetic genes for secondary metabolites: new global regulators and expanding roles for cluster-situated regulatory genes

As a broad generalisation, antibiotic biosynthetic gene clusters depend on regulatory genes located within the cluster, which are themselves directly regulated by many higher level regulatory proteins, encoded by genes such as the developmental *bld* genes scattered around the chromosome ^100^. New regulators continue to be identified: a TetR-like protein specified by Sco3201 has been found to bind to the promoters of several known developmental and antibiotic biosynthetic genes ^101^, and the *abrC* cluster (Sco4596–8) encoding a response regulator (AbrC3) and two histidine protein kinases has been shown to influence secondary metabolism ^102, 103^. An accumulation of papers has shown that the promoter of the *actII-orf4* gene that activates the ACT biosynthetic genes of *S. coelicolor* binds at least eight, and possibly as many as 12, regulatory proteins ^100^, a number to which AbrC3 has been added ^103^. A different scenario was observed in the case of the novobiocin biosynthetic genes when heterologously expressed in *S. coelicolor*: a promoter of these biosynthetic genes (instead of a regulatory gene) was a target for as many as 11 host-specified proteins and the cluster-situated NovG regulatory protein ^104^. Only one of these 11 proteins, AbsC, is also known to bind to *actII-orf4*.

Regulatory genes such as *actII-orf4* and *novG* that are situated within biosynthetic clusters were previously called “pathway specific”, but numerous observations have shown that they may have targets outside the cluster, so their renaming as “cluster-situated regulators” (CSRs) by 105 has proved prescient. In a striking recent example involving *Streptomyces avermitilis*, a CSR belonging to a class with PAS-LuxR-like domains was shown to interact with genes for different pathways and with many other promoters for housekeeping genes ^106^.

### Small-molecule ligands for proteins that regulate antibiotic production: feedback and feedforward regulation and crosstalk within and between organisms

Some of the proteins regulating antibiotic production interact with known small-molecule ligands associated with different aspects of primary metabolism ^100^. Another widespread scenario is the involvement of diffusible GBL and related signal molecules as the ligands of a subfamily of TetR-like regulators, like the A-factor/ArpA pair of *S. griseus* (see above). This may operate differently in different species and different pathways. In *S. venezuelae*, the SVB1 GBL receptor JadR3 has complex interactions with the promoters of both its own biosynthetic genes and jadomycin biosynthetic genes. This results in an integrated feedback and feedforward regulatory scheme that accounts for both the onset of the major period of jadomycin production and its subsequent down-regulation ^66^. In *S. coelicolor*, genome-wide ChIP-seq analysis has revealed an extensive network of regulatory interactions centred on ScbR (GBL receptor) and ScbR2 (pseudo-GBL receptor that binds antibiotics) ^107, 108^.

A major development in the regulation of antibiotic production concerns the activities of secondary metabolites themselves as regulatory ligands ^65, 100^. In one recent example from *Streptomyces antibioticus*, the spirotetronate chlorothricin and some of its biosynthetic intermediates act as regulatory ligands for the CSR ChlF1 ^109^. In another case involving the aminocoumarin cacibiocin, a repressor of the biosynthetic genes, CabR, loses its DNA-binding activity in the presence of the end product or other aminocoumarins ^110^.

Internal cross-pathway regulation mediated by end products has been subjected to closer analysis following the discovery of crosstalk between the chloramphenicol and jadomycin biosynthetic pathways in *S. venezuelae.* JadR1, the main activator of the jadomycin gene cluster, was implicated in repressing chloramphenicol biosynthesis ^111^, but a recent further analysis of this situation revealed that cross-regulation, though still observed, was markedly different when the experiments were done on a different medium ^112^. It is not yet known whether these differences involve *jadX*, another newly identified regulatory gene in the jadomycin cluster ^113^. Knocking out *jadX* prevented the well-known induction of jadomycin production by stress conditions while at the same time putting chloramphenicol biosynthesis under the control of these stresses, and it was shown that both jadomycins and chloramphenicol could interact with JadX ^113^. Interestingly, genes for JadX-like proteins are present in some other antibiotic biosynthetic clusters ^113^.

In *S. coelicolor,* ScbR2, a protein closely similar to GBL-binding proteins, has been found to interact with jadomycins from *S. venezuelae* (and other heterologously produced angucyclines) to relieve the ScbR2-mediated repression both of the global regulatory gene *adpA* and of *redD*, encoding an activator of the RED pathway genes ^114^. Thus, *S. coelicolor* can respond to the presence of another species by undergoing developmental changes and switching on antibiotic production. Another example of such a response has been documented for lidamycin biosynthesis in *Streptomyces globisporus,* involving a widely conserved TetR-like global activator of antibiotic production, AtrA ^107^. AtrA directly activates lidamycin biosynthetic genes, but its activity is prevented by binding either a lidamycin biosynthetic intermediate, heptaene, or heterologously produced ACT ^107^. Inter-species interactions affecting antibiotic production are proving to be widespread among streptomycetes: a large-scale study of inter-organism interactions revealed many cases of production of secondary metabolites induced by growth close to another species ^115, 116^, and another study found that low levels of lincomycin induced ACT production by *S. coelicolor* ^117^. It will be interesting to see whether ScbR2-, JadR1-, and JadX-like proteins are often involved in such crosstalk, or even in some of the more divergent interactions of streptomycetes with other microbes that continue to emerge from co-culture studies (for recent examples, see 118– 121).

## A high-resolution global survey of gene expression in *S. coelicolor* at different growth stages and the importance of translation-level regulation

Through the use of RNA sequencing to analyse the global transcription profile, and ribosome profiling (ribo-seq) to analyse translation, a near-comprehensive gene expression picture of *S. coelicolor* at different stages of growth in liquid culture has been obtained ^122^. This survey identified most of the transcription start sites in the entire genome, including those for 230 small RNAs. Amazingly, about 21% of protein-coding RNAs are leaderless (and lack conventional ribosome-binding sites). Moreover, clear changes were seen in translation rates between different growth stages, including increased efficiency of translation of mRNAs for major CSR genes at the end of the main growth phase but decreased efficiency for the biosynthetic genes that they control. It is not clear whether these changes reflect different codon usage, a relevant question given that several of the CSR genes for antibiotic production contain the very rare codon TTA ^65, 100^. The protein-level expression of TTA-containing genes is usually dependent on the *bldA* gene, which encodes the cognate tRNA; abundance of this tRNA increases on the transition into stationary phase ^123, 124^.

In an interesting counterpoint to the *bldA* story, the TTA codon in *ccrA,* the CSR gene controlling cephamycin C and clavulanic acid biosynthesis in *S. clavuligerus*, does not make the production of CcrA *bldA*-dependent ^125^. It has now turned out that many, but not all, TTA-containing genes in *S. clavuligerus* display the same anomaly ^126^. It would perhaps be worth examining the *bldA* dependence of these genes when introduced into *S. coelicolor* and of TTA-containing genes from *S. coelicolor* upon introduction into *S. clavuligerus*.

## Technological advances

The last few years have seen steps forward in the development and application of diverse technologies for the study and exploitation of streptomycetes. The first systems for applying genome editing to streptomycetes will soon accelerate the construction of diverse kinds of mutant ^127– 130^, while the discovery of a CRISPR-Cas system in *S. avermitilis* may open up new approaches ^131^. Along with new strategies for the cloning and deletion of very large segments of *Streptomyces* DNA, sufficient to encompass almost all antibiotic biosynthetic gene clusters ^132, 133^, procedures are in place to exploit the ever-growing numbers of uncharacterised clusters emanating from genome sequencing, along the lines summarised by 134 and 135. Bioinformatic tools have been developed to predict the nature of the products of such clusters ^136^. One component of systems-based exploitation will be a panel of strong promoters, such as 20 promoters significantly stronger than the widely used ermE* that have been characterised in *S. albus* G ^137^. Another promoter survey has identified promoters with very stable expression levels at different growth stages in two strains of *S. coelicolor* A3(2) and in other species ^138^. These are likely to be valuable standards in transcription studies.

## Concluding remarks

The rapid recent progress summarised in this article seems set to accelerate further, particularly in light of the imminent application of powerful additions to the techniques available for *Streptomyces* research. Research targets will include further elucidation of the ligands that determine the activity of regulatory proteins, the ways in which developmentally important proteolysis is controlled, the search for the molecular factors involved in symbiotic associations, and the structural and functional characterisation of the DivIVA-centred polarisome that determines tip growth. In the course of such studies, an integrated picture of these diverse aspects of *Streptomyces* biology will begin to emerge. It is likely that this will require increasing input from computational biologists. The accumulating understanding of the biology of these key antibiotic-producing organisms can be expected to facilitate the development of new therapeutic agents in the global struggle against antibiotic-resistant pathogens.

## Abbreviations

ACT, actinorhodin; ChIP-seq, chromosome immunoprecipitation sequencing; CSR, cluster-situated regulator; GBL, gamma-butyrolactone; RED, undecylprodiginine.
